# Supplementary material for: Both maternal IFNγ exposure and acute prenatal infection with Toxoplasma gondii activate fetal hematopoietic stem cells
Source: EMBO J. 2023 Jun 1;42(14):e112693. doi: 10.15252/embj.2022112693 (PMC10350822; doi:10.15252/embj.2022112693)
Supplement: Supplementary file 1 — Expanded View Figures PDF [file EMBJ-42-e112693-s002.pdf]

Expanded View Figures

Figure EV1. Maternal infection with *Toxoplasma gondii* modulates fetal growth and hematopoietic development.

A Visual comparison of crown-rump length from E16.5 fetuses from saline and RH infected mothers, as shown in Fig 1A.
B Fraction of viable (or "non-resorbed") fetuses per litter observed in E16.5 litters following saline or infection with Pru or RH; n = 4 litters/condition.
C Schematic of hematopoietic stem and progenitor (HSPC) cell hierarchy and surface markers.
D Representative gating strategy for fetal liver Tom+ HSCs and GFP+ drHSCs at E16.5.
E–L Frequency of (E) HSPCs (F) LT-HSCs, (G) ST-HSCs, (H) Tom+ HSCs, (I) GFP+ drHSCs, (J) MPP2, (K) MPP3, (L) MPP4 in E16.5 fetuses following saline or maternal infection with Pru or RH as shown in Fig 1A. n = 9–15 fetuses from 3 litters per each condition.
M Total cellularity of E16.5 fetal liver cells following saline or infection with Pru or RH.
N Total cellularity of CD45+ cells in fetal liver at E16.5 following saline or infection with Pru or RH. For E and N, each dot represents results from an individual fetus, 4 litters were analyzed per each condition. Data information: For all analysis above bars represent mean.

Data analyzed by one-way ANOVA with Tukey's test. *$P \leq 0.05$; **$P \leq 0.01$; ***$P \leq 0.001$; ****$P \leq 0.0001$.
Source data are available online for this figure.

© 2023 The Authors The EMBO Journal 42: e112693 | 2023 17 of 16

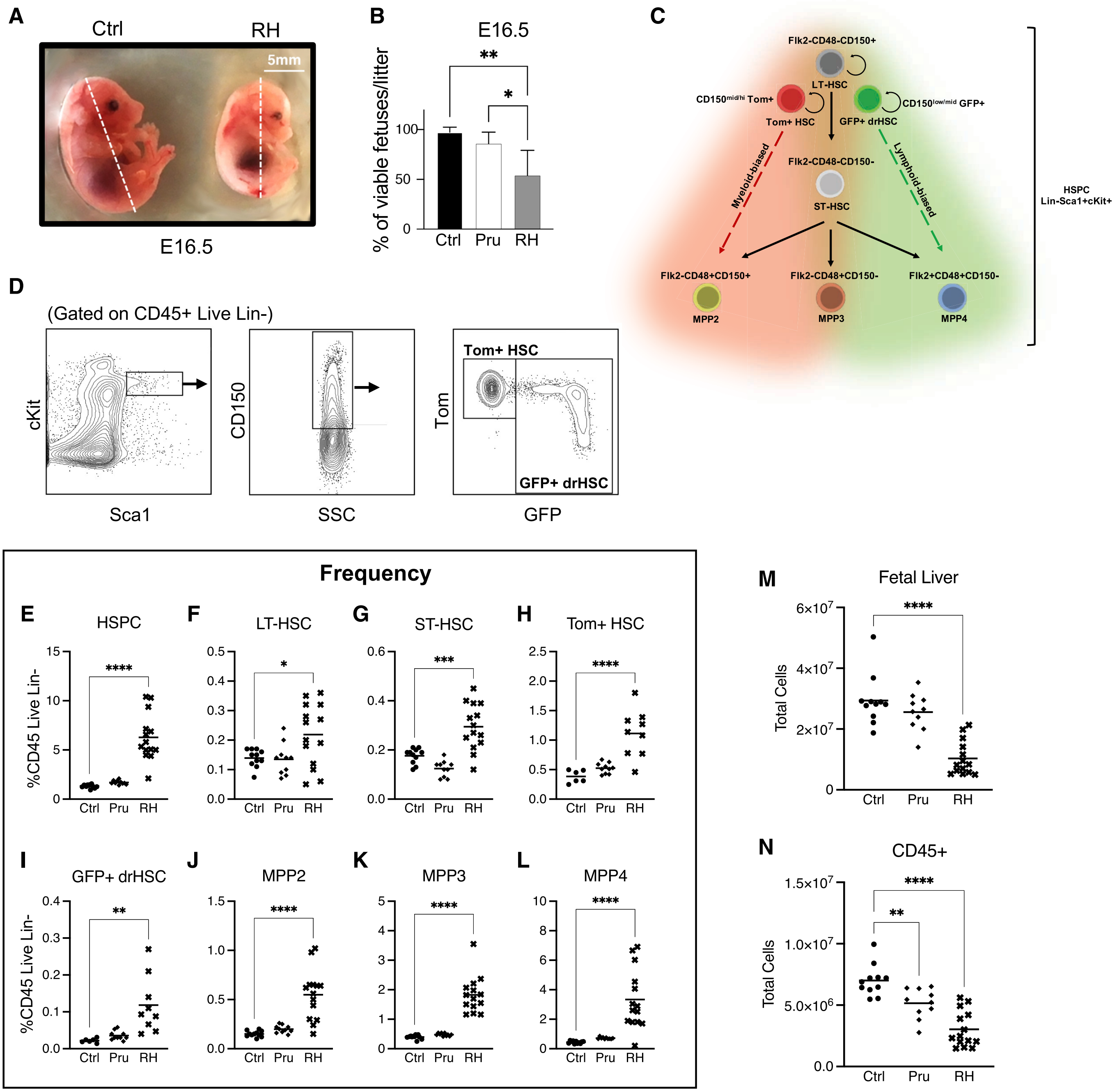

Figure EV1.

 © 2023 The Authors

Figure EV2. Maternal infection with *Toxoplasma gondii* leads to lasting changes in BM reconstitution of fetal HSCs.

A–M Bone Marrow (BM) chimerism of (A) HSPCs, (B) LT-HSCs, (C) ST-HSCs, (D) MPP2, (E) MPP3, (F) MPP4, (G) granulocyte macrophage progenitors (GMP), (H) megakaryocyte progenitors (MkP), (I) erythroid progenitors (EP), (J) granulocyte/macrophages (GM), (K) common lymphoid progenitors (CLP), (L) B-cells, and (M) T-cells in primary recipients of Tom+ HSCs or GFP+ drHSCs at 18-week post-transplantation. *n* of mice is shown as the numerator in Fig 2B.

N–Z Bone Marrow (BM) chimerism of (N) HSPCs, (O) LT-HSCs, (P) ST-HSCs, (Q) MPP2, (R) MPP3, (S) MPP4, (T) granulocyte macrophage progenitors (GMP), (U) megakaryocyte progenitors (MkP), (V) erythroid progenitors (EP), (W) granulocyte/macrophages (GM), (X) common lymphoid progenitors (CLP), (Y) B-cells, and (Z) T-cells in secondary Tom+ HSC or GFP+ drHSC transplant recipients at 18-week post-transplant. *n* of mice is shown as the numerator in Fig 2K.

Data information: For all analyses above, bars represent mean ± SD. Statistical significance was determined by one-way ANOVA with Tukey's test. *$P \leq 0.05$; **$P \leq 0.01$; ***$P \leq 0.001$. No reconstitution was present for the secondary recipients of the GFP+ drHSC under RH condition.
Source data are available online for this figure.

© 2023 The Authors

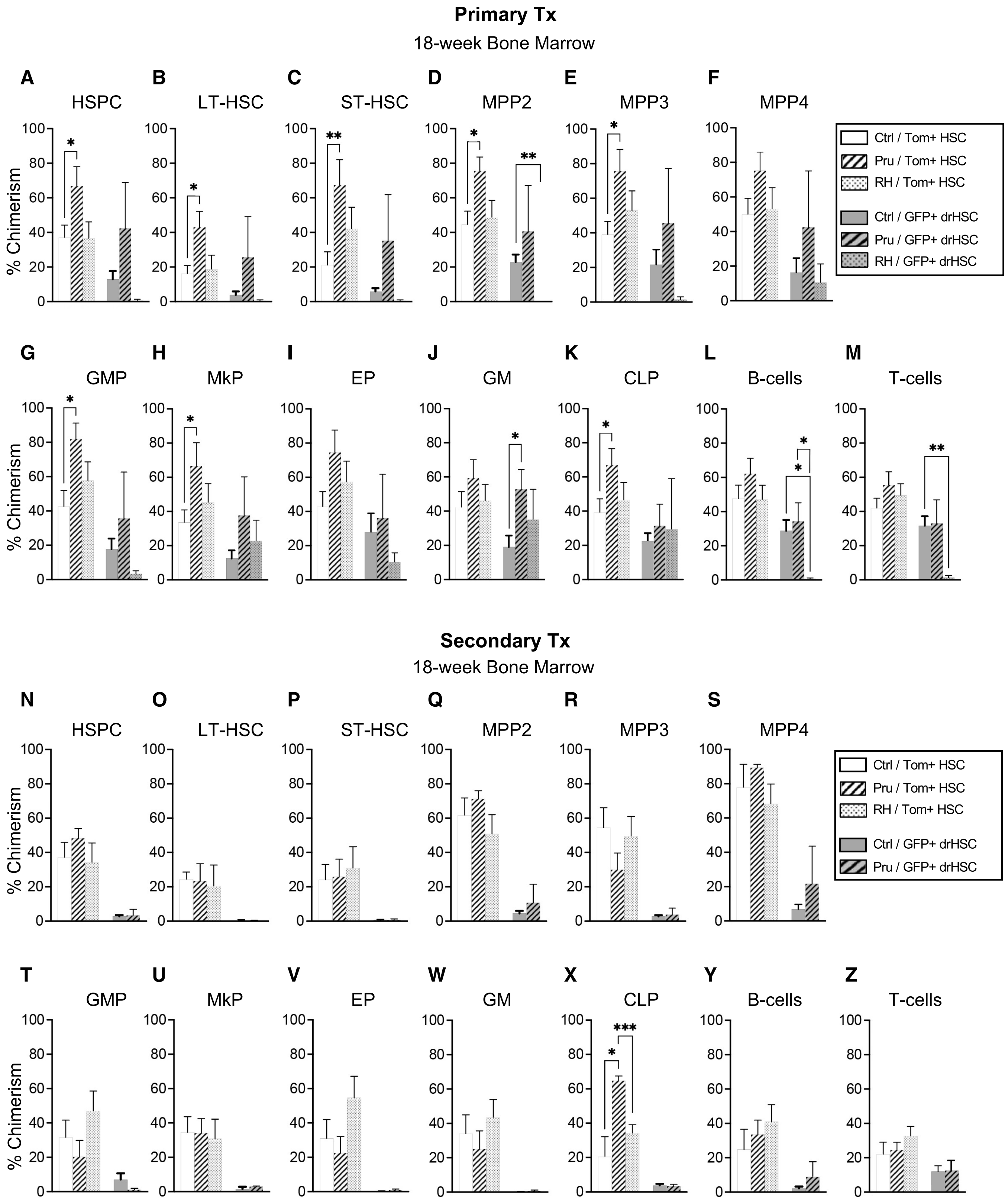

Figure EV2.

 © 2023 The Authors

Figure EV3. Bone marrow chimerism from IFNγ exposed fetal HSCs following transplantation.

A–M Bone Marrow (BM) chimerism of (A) HSPCs, (B) LT-HSCs, (C) ST-HSCs, (D) MPP2, (E) MPP3, (F) MPP4, (G) granulocyte macrophage progenitors (GMP), (H) megakaryocyte progenitors (MkP), (I) erythroid progenitors (EP), (J) granulocyte/macrophages (GM), (K) common lymphoid progenitors (CLP), (L) B-cells, and (M) T-cells in primary recipients of Tom+ HSCs or GFP+ drHSCs at 18-week post-transplantation. *N* of mice is shown as the numerator in Fig 5A.

N–Z Bone Marrow (BM) chimerism of (N) HSPCs, (O) LT-HSCs, (P) ST-HSCs, (Q) MPP2, (R) MPP3, (S) MPP4, (T) granulocyte macrophage progenitors (GMP), (U) megakaryocyte progenitors (MkP), (V) erythroid progenitors (EP), (W) granulocyte/macrophages (GM), (X) common lymphoid progenitors (CLP), (Y) B-cells, and (Z) T-cells in secondary recipients of Tom+ HSC or GFP+ drHSCs at 18-week post-transplantation. *N* of mice is shown as the numerator in Fig 5J.

Data information: For all experiments above, bars represent mean ± SD. Statistical significance was determined by one-way ANOVA with Tukey's test. $*P \leq 0.05$; $**P \leq 0.01$; $***P \leq 0.001$.
Source data are available online for this figure.

© 2023 The Authors

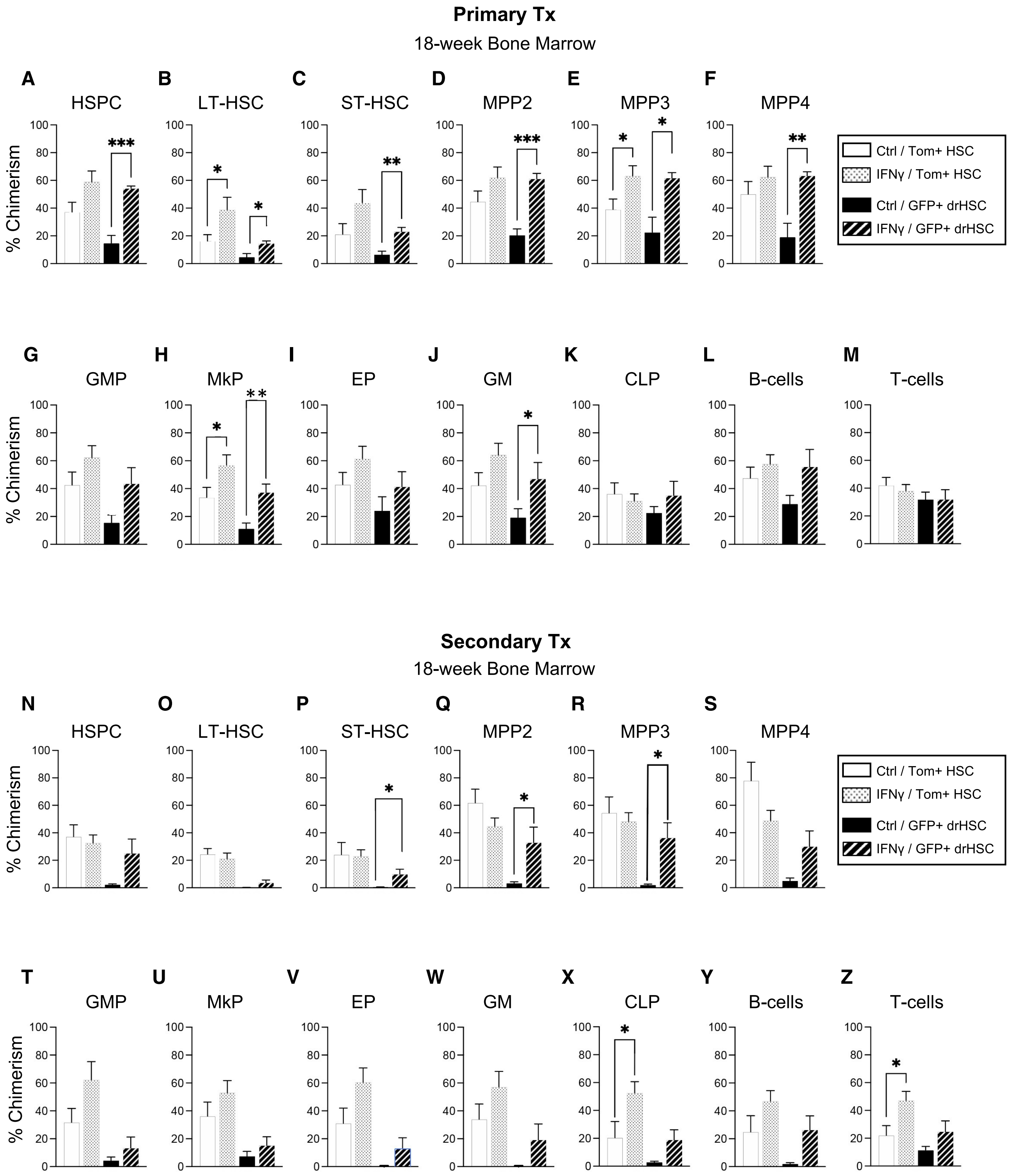

Figure EV3.

© 2023 The Authors

Figure EV4. Role of IFNγ and IFNγR in fetal response.

A Comparison of IFNγ cytokine in E15.5 fetal amniotic fluid between IFNγR +/− and −/− pups from either IFNγR +/− or −/− dams following saline injection.
B Comparison of IFNγ cytokine in E15.5 fetal amniotic fluid between IFNγR +/− and −/− pups from either IFNγR +/− or −/− dams following IFNγ injection.
C Comparison of IFNγ cytokine in E15.5 fetal liver supernatant between IFNγR +/− and −/− pups from either IFNγR +/− or −/− dams following saline injection.
D Comparison of IFNγ cytokine in E15.5 fetal liver supernatant between IFNγR +/− and −/− pups from either IFNγR +/− or −/− dams following IFNγ injection.
E Comparison of IFNγ cytokine in E15.5 maternal serum between IFNγR +/− and −/− dams following saline or IFNγ injection.
F Population changes in HSCs and MPPs in IFNγR +/− and −/− pups from either IFNγR +/− or −/− dams following IFNγ injection.

Data information: For amniotic fluid and fetal liver supernatant, n = 7–10 fetuses from 2–4 litters per condition. For maternal serum, n = 2–4 dams per condition. For all experiments above, bars represent mean ± SD. Statistical significance was determined by two-way ANOVA. **$P \le 0.01$; ****$P \le 0.0001$. N = 10–14 fetuses from at least 3 litters/condition.
Source data are available online for this figure.

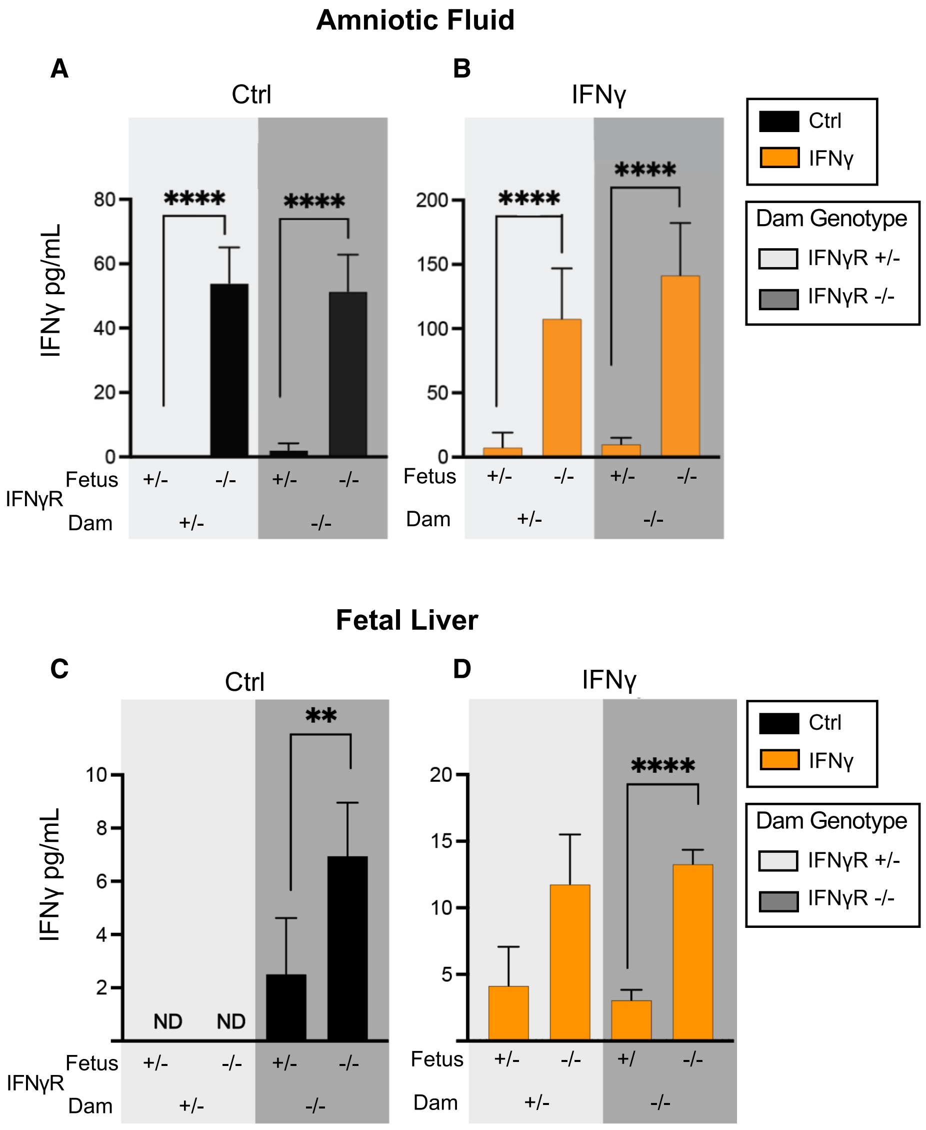

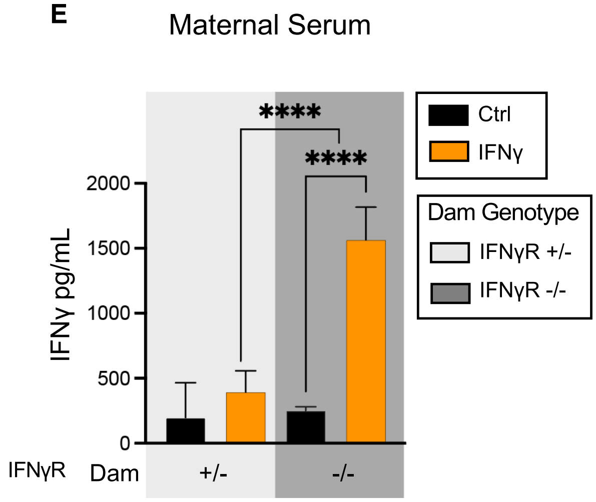

F

IFNγR		HSCs	MPPs
**Dam**	**Fetus**		
+/-	+/-	Up	Up
+/-	-/-	No change	Up
-/-	+/-	No change	Up
-/-	-/-	No change	No change

Figure EV4.

© 2023 The Authors
